# Identification and evaluation of a six-lncRNA prognostic signature for multiple myeloma

**DOI:** 10.1007/s12672-024-01064-3

**Published:** 2024-06-03

**Authors:** Lu Xu, Zhihao Xie, Huanlin Jiang, Erpeng Wang, Min Hu, Qianlei Huang, Xinbao Hao

**Affiliations:** 1 Department of Hematology, The First Affiliated Hospital of Hainan Medical College, Haikou, 570102 China; 2 Tsinghua University, School of Medicine, Beijing, 100084 China; 3 City Hospital of Qingdao, Qingdao, 266011 China; 4 Nanfang Medical University, Guangzhou, 510515 China; 5 Department of Hematology, Roswell Park Comprehensive Cancer Center, Buffalo, NY 14263 USA

**Keywords:** Long noncoding RNA, Multiple myeloma, Prognosis, Biomarker, Competing endogenous RNA, ceRNA

## Abstract

**Purpose:**

Multiple myeloma (MM) is the second most common hematologic malignancy, and there is no cure for this disease. This study aimed to explore the prognostic value of long noncoding RNAs (lncRNAs) in MM and to reveal related immune and chemotherapy resistance mechanisms.

**Methods:**

In this study, lncRNA profiles from the Multiple Myeloma Research Foundation (MMRF) and Gene Expression Omnibus (GEO) databases were analyzed to identify lncRNAs linked to MM patient survival. A risk assessment model stratified patients into high- and low-risk groups, and survival was evaluated. Additionally, a triple-ceRNA (lncRNA–miRNA–mRNA) network was constructed, and functional analysis was performed. The research also involved immune function analysis and chemotherapy drug sensitivity assessment using oncoPredict and the GDSC dataset.

**Results:**

We identified 422 lncRNAs significantly associated with overall survival in MM patients and ultimately focused on the 6 with the highest prognostic value. These lncRNAs were used to develop a risk score formula that stratified patients into high- and low-risk groups. Kaplan–Meier analysis revealed shorter survival in high-risk patients. We integrated this lncRNA signature with clinical parameters to construct a nomogram for predicting MM prognosis. Additionally, a triple-ceRNA network was constructed to reveal potential miRNA targets, coding genes related to these lncRNAs and significantly enriched pathways. Immune checkpoint gene expression and immune cell composition were also analyzed in relation to the lncRNA risk score. Finally, using the oncoPredict tool, we observed that high-risk patients exhibited decreased sensitivity to key MM chemotherapeutics, suggesting that lncRNA profiles are linked to chemotherapy resistance.

**Supplementary Information:**

The online version contains supplementary material available at 10.1007/s12672-024-01064-3.

## Introduction

Multiple myeloma (MM), a bone marrow-resident hematological malignancy of plasma cells, has remained largely incurable despite dramatic improvements in patient outcomes in the era of myeloma-targeted and immunomodulatory agents [1]. MM is caused by several genetic factors, including changes in chromosomal copy number [2, 3], aberrant gene methylation [4, 5], and dysregulated gene expression [6, 7]. Considerable progress has been made in the diagnosis and treatment of MM in the last several decades. However, the current prognostic factors for patients with MM do not meet clinical needs, making it necessary to identify novel biomarkers that are sensitive and accurate for better predicting overall survival.

Long noncoding RNAs (lncRNAs), which typically exceed 200 nucleotides in length, are a distinct class of RNAs known for their lack of protein-coding potential [8]. Recent studies have highlighted lncRNAs as pivotal contributors to various complex biological processes, including cellular proliferation, cell cycle progression, and survival mechanisms [9]. Notably, lncRNAs have been implicated as modulators in the oncogenesis process that influence invasion and metastasis rates across several cancer types [10, 11]. LncRNAs, also known for their regulatory roles in gene expression, have emerged as key factors in sustaining the stemness of cancer stem cells, thereby influencing chemo- and immune-resistance mechanisms in MM [12]. Furthermore, lncRNAs contribute to chemoresistance by modulating drug efflux, DNA damage repair, the cell cycle, apoptosis, epithelial–mesenchymal transition (EMT), and various signaling pathways [13]. The tumor microenvironment (TME) in MM is intricately regulated by lncRNAs, which play roles in promoting tumor immunosuppression, thereby impacting the efficacy of immunotherapies [14]. lncRNAs regulate miRNAs, signaling pathways, and proteins, thereby influencing both pro- and antiapoptotic mechanisms and autophagy and contributing to multidrug resistance in cancer cells [15]. Moreover, immune-related lncRNAs have been identified as critical regulators of immune cell-specific gene expression and thus may affect immunotherapy resistance [16].

Despite these advances, the biological functions and prognostic significance of many lncRNAs remain largely unexplored. A particularly intriguing aspect is the role of numerous lncRNAs as competing endogenous RNAs (ceRNAs), which regulate the expression of protein-coding genes by sharing common microRNA response elements (MREs) [17]. This study focused on investigating the predictive value of lncRNAs in MM patients and aimed to explore the role of lncRNAs in immune and chemotherapy resistance mechanisms in MM, considering their potential as both biomarkers and therapeutic targets, and investigated their roles within the ceRNA network.

## Materials and methods

### Data processing and computational analysis

**Fig. 1:**
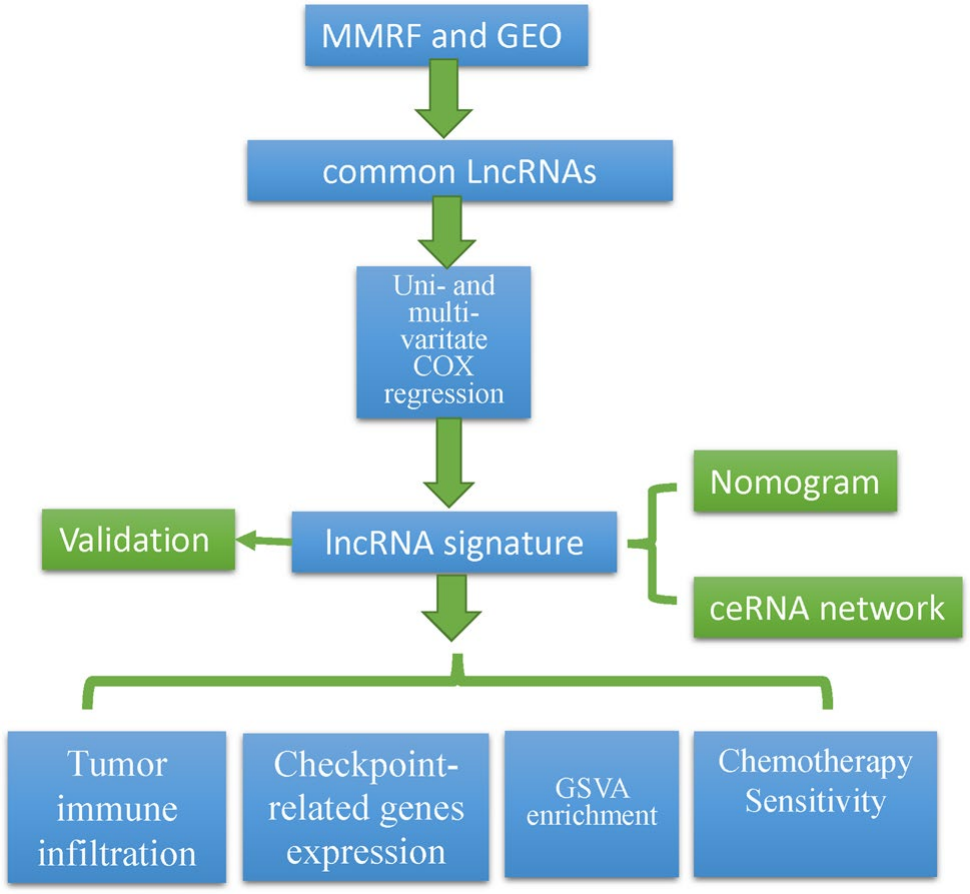
Workflow for the identification and functional evaluation of lncRNAs in MM

Figure 1 shows the overall workflow of this study. The transcriptome data (RNA expression profiles) were downloaded from the Multiple Myeloma Research Foundation (MMRF, https://xenabrowser.net) and Gene Expression Omnibus (https://www.ncbi.nlm.nih.gov/geo/) data portals (dated to November 11, 2022).

### Identification of lncRNAs related to patient prognosis

In this study, sample selection involved an initial filtration step, wherein patients lacking comprehensive survival data were excluded from analysis. Subsequently, 858 samples from the MMRF datasets were designated the training set, and 559 samples from the GSE24080 dataset constituted the validation set. The clinical and demographic characteristics of the cohorts are shown in Table S1 and Table S2. Notably, statistical parity was observed between the two datasets. The potential prognostic value of candidate lncRNAs in MM was first assessed via univariate Cox proportional hazards regression analysis, aiming to identify lncRNAs correlated with overall survival [19].

### Establishment and validation of the risk formula

In the subsequent stage of our study, lncRNAs identified in the preceding step were incorporated into a multivariate Cox proportional hazard model within the training set to compute the coefficients, which were used to develop the risk assessment formula. This formula was used to calculate risk scores for each patient under study. Patients were stratified into high- and low-risk groups based on the median risk score. Survival analysis, particularly overall survival (OS), was conducted between these groups using the Kaplan‒Meier method and the log-rank test via the 'survival' package in R. To assess the predictive accuracy of our model, a time-dependent receiver operating characteristic (ROC) curve was constructed (software version 1.0.3). The graphical representations of these analyses were generated using ‘ggplot2’ (version 2.2.1) and ‘ggfortify’ (version 0.4.1). Data processing and analysis were comprehensively conducted using Excel 2010 and R (version 4.3.0). The function cph was applied with the R package rms to fit the data and construct a nomogram.

### LncRNA function exploration and ceRNA network construction

In this research, the functional attributes of the identified lncRNAs were investigated through the construction of a triple ceRNA (lncRNA–miRNA–mRNA) network. The sequences of these lncRNAs, sourced from the Ensembl database, were entered into the miRDB database for the prediction of their miRNA targets. Subsequently, the associated coding genes were identified utilizing a combination of databases: miRDB, miRTarBase, and TargetScan. The intricate triple-ceRNA network was then meticulously visualized and constructed using Cytoscape v3.5.1. Furthermore, pathway enrichment of each sample was analyzed by the GSVA and clusterProfiler R packages. A significance threshold (cutoff P value of 0.05) was set to ensure the robustness of the analytical outcomes.

### Immune function correlation analysis

The expression values of 20 immune checkpoint-related genes were extracted. The R package ggplot2 was used for analysis, and the results are presented in the illustration. The significance of differences between two groups was compared through the Wilcoxon test.

quanTIseq is based on a deconvolution algorithm that uses RNA-seq data from bulk samples to predict the composition of different types of immune cells in tumor samples [18]. We used quanTIseq to predict the composition of different types of immune cells in tumor samples using bulk RNA-seq data.

### Chemotherapy sensitivity analysis

Chemotherapy sensitivity analysis using the oncoPredict R package and the Genomics of Drug Sensitivity in Cancer (GDSC) dataset represents a comprehensive approach that significantly enhances the accuracy and efficacy of cancer treatment response prediction [19]. This method increases the correlation between the actual and predicted chemotherapy drug sensitivity results by integrating heterogeneous drug sensitivity data, particularly improving the agreement for targeted therapies such as kinase inhibitors [20]. Predictions of drug sensitivity for samples from the MMRF dataset were made using the oncoPredict R package with v2 of Genomics of Drug Sensitivity in Cancer (GDSC) as the training set.

### Cell culture and transfection

The multiple myeloma cell line NCI-H929 was obtained from the American Type Culture Collection (ATCC). All cells were cultured in RPMI 1640 medium supplemented with 10% fetal bovine serum (Gibco), 100 μg/mL streptomycin (Invitrogen), and 100 U/mL penicillin (Invitrogen) in a humidified incubator containing 5% CO_2_ at 37 °C.

The lentiviruses containing TMPO_AS1 shRNA CTTAGACGCCGATAAGGGAC, SNHG17 shRNA GATTGTCAGCTGACCTCTGTCCTGT and negative control shRNA UUCUCCGUUCGUGUCACGTTT were designed and constructed by Creative Biolabs (NY, USA).

NCI-U929 cells were transduced with lentivirus containing shRNAs targeting TMPO_AS1 and SNHG17 in the presence of 5 mg/mL polybrene and selected with 5 μg/mL puromycin (Thermo Fisher, USA) to establish stable knockdown cells. Cell transfection and lentiviral transduction were performed according to the manufacturer’s instructions.

### RNA extraction and RT‑qPCR

TRIzol (Invitrogen, USA) was used to extract total RNA from cultured cells according to the manufacturer’s instructions. cDNA synthesis kits provided by Thermo Fisher (USA) were utilized to generate cDNAs through reverse transcription. SYBR Green Master Mix (Invitrogen, USA) was used for quantitative PCR. The relative expression levels of TMPO_AS1 and SNHG17 mRNA were normalized to that of GAPDH, which was used as an internal control. The relative expression was calculated by the 2^–ΔΔCt^ method.

The following primers from IDT DNA (Iowa, USA) were used: TMPO_AS1 (F: CCAGACCCGGACACAAAAGA; R: CTGCGTTTCTACCTCCTCTCG), SNHG17 (F: AGGGGAAGCAAGGTGAAAGT; R: ATCCCAGATCACCAACTCCA), and GAPDH (F: AGGTCGGAGTCAACGGATTT; R: TGACGGTGCCATGGAATTTG) were used.

### Cell proliferation assay

For cell viability assessments using PrestoBlue™ HS, cells were seeded in 96-well plates and incubated at 37 °C. If needed, the cells were treated with chemotherapy reagents before the assay. PrestoBlue™ HS was added at 1/10th volume to each well and incubated for a minimum of 30 min at 37 °C in the dark. Viability was measured via fluorescence (excitation at 560 nm, emission at 590 nm).

### Colony formation assay

In the simplified colony-forming assay in soft agar, NCI-H929 cells are embedded in a mixture of Noble agar and media and layered between solidified agar in 6 cm plates. After incubation at 37 °C for 2–3 weeks, visible colonies were counted to assess cell survival and proliferative capacity compared to those of untreated controls, providing insights into the effectiveness of the cytotoxic treatment. This method evaluates the long-term impact of treatments on hematopoietic cancer cells and is crucial for therapeutic research [21, 22].

### Statistical analysis

All statistical analyses were performed in R (v3.6.3) and GraphPad Prism (version 8.0). Statistical significance was assessed using an independent sample t-test or the Wilcoxon rank sum test for expression data. In correlation analysis, the Pearson or the Spearman correlation coefficient was used to evaluate the correlation according to the samples’ distribution. The survival R package (v3.2-8) was used for statistical survival data analysis. Three independent replicates were performed for all cell and qPCR experiments. Data were presented as the mean ± standard deviation (SD). p < 0.05 was considered statistically significant.

## Results

### Identification of a lncRNA signature for prognosis prediction

Through univariate Cox regression analysis of the total dataset, 422 lncRNAs were identified as significantly associated with overall survival. Among these, the 15 lncRNAs with the lowest P values were subjected to further analysis. The risk coefficients for these selected lncRNAs were computed employing a multivariable Cox proportional hazards model. The resulting risk score formula was established as follows: 0.22*TMPO_AS1 + 0.20*DLG3_AS1 + 0.10*SH3RF3_AS1 + 0.15*DSCR8 + (-0.10)*DSCR4 + 0.29*SNHG17.

**Fig. 2:**
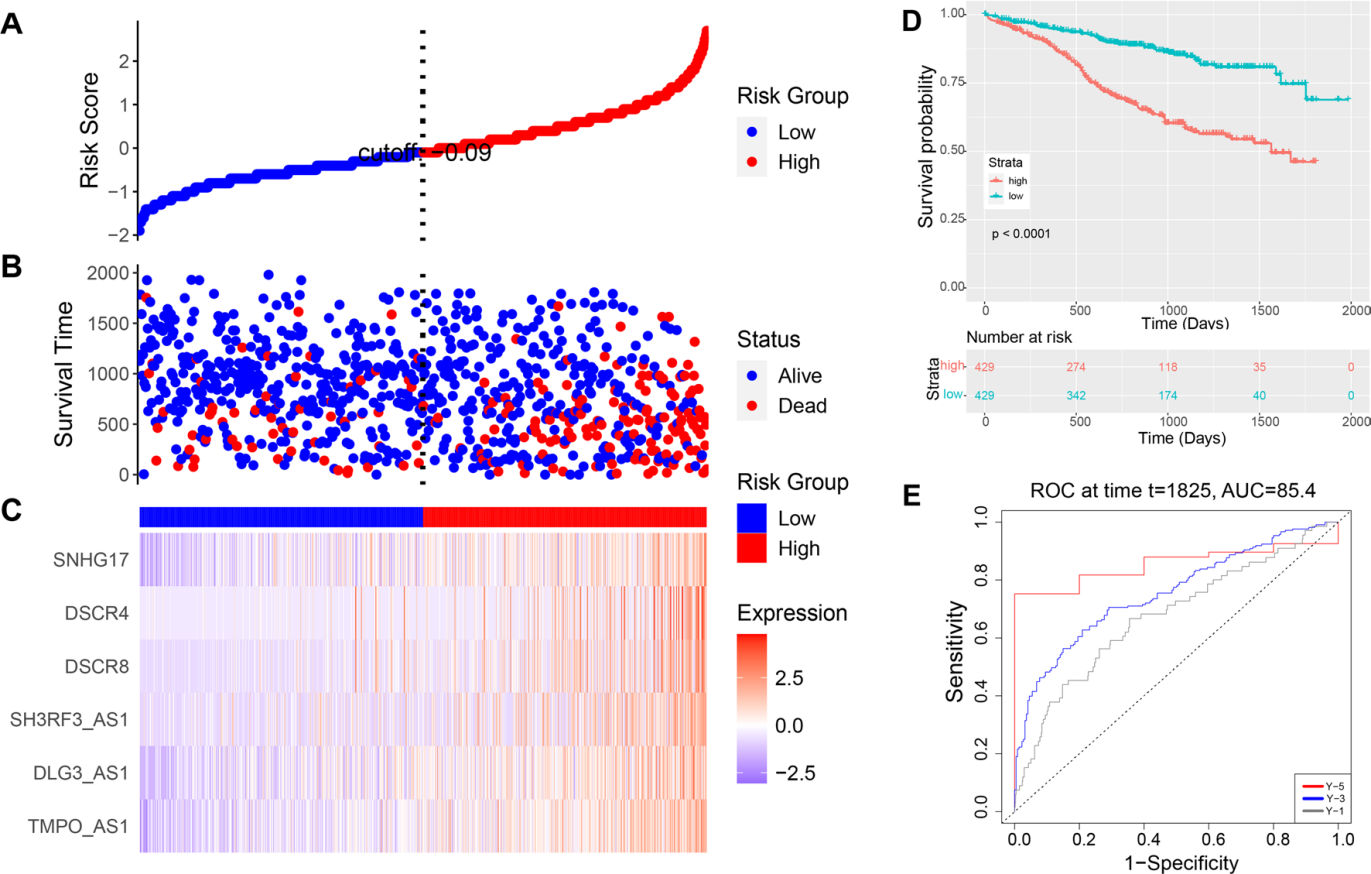
Risk scores for multiple myeloma patients based on the lncRNA signature. **A** Patients were divided into two groups according to their risk scores. **B** Distribution of patient survival time. **C** lncRNA signature expression levels in each patient. **D** Analysis of patient survival according to lncRNA risk group. **E** ROC analysis of the prognostic value of the lncRNAs

Subsequently, risk scores were calculated for each patient within the training set, leading to their stratification into two groups based on the median risk score (Fig. 2A). The distribution of patient survival times is shown in Fig. 2B. The expression profiles of the six lncRNAs in the two groups are presented in Fig. 2C. Survival analysis, performed using the Kaplan–Meier method and the log-rank test, revealed a significantly shorter survival duration in patients with higher risk scores (P < 0.001), as depicted in Fig. 2D. A negative correlation was observed between survival time and the risk score in our analysis. The prognostic value of the 6-lncRNA signature was evaluated through receiver operating characteristic (ROC) analysis (Fig. 2E). The area under the curve (AUC) was determined to be 0.854.

### Validation of the prognostic model of the lncRNA signature and nomogram integration

**Fig. 3:**
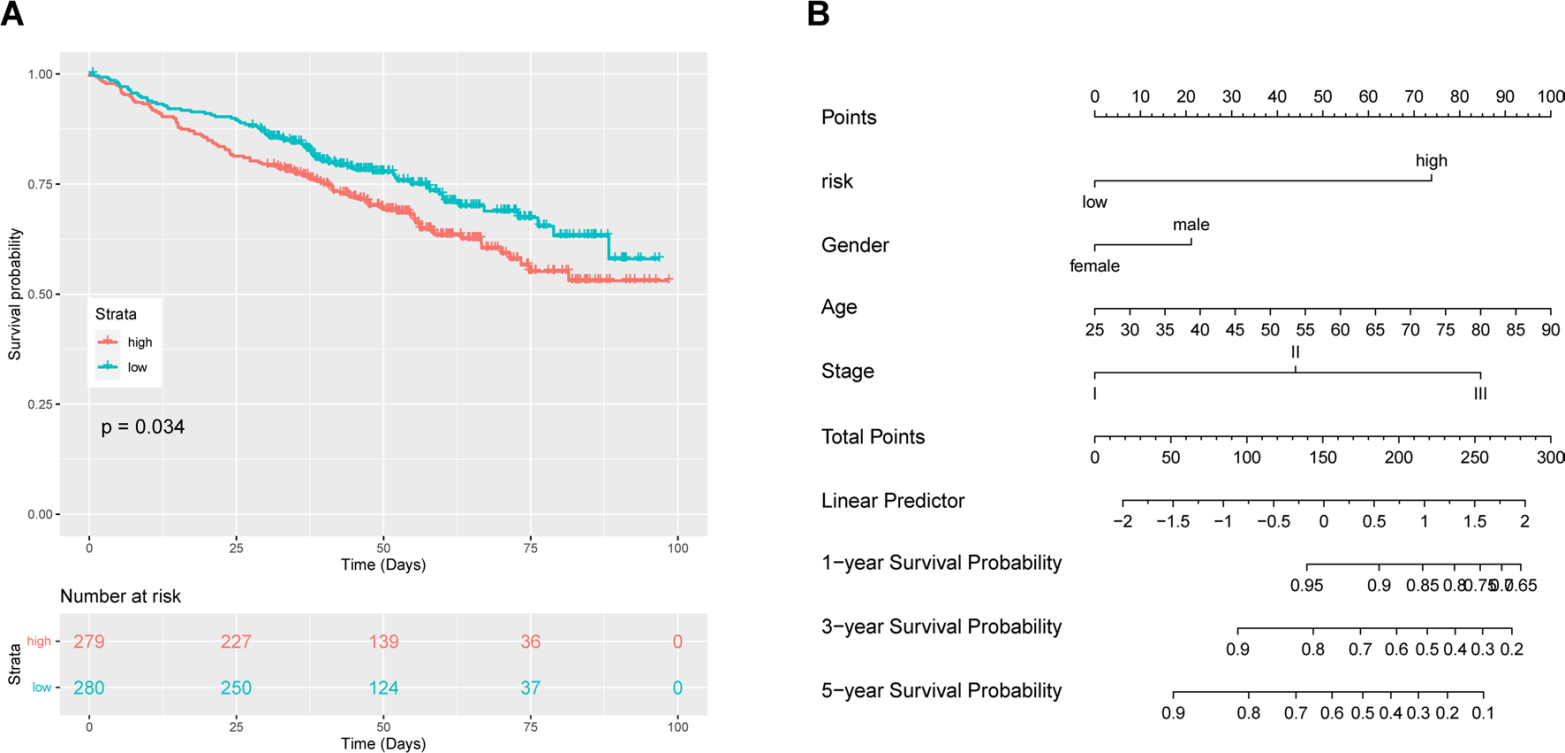
Validation of the lncRNA signature for prognostic evaluation and nomogram construction **A** Risk assessment of lncRNAs was used for prognosis analysis for patients in the validation cohort. **B** A nomogram containing risk scores and clinical data was constructed to assess patient outcomes

In the validation phase of the study, a cohort comprising 559 samples from the validation set was utilized to ascertain the efficacy of the developed lncRNA prognostic models. Figure 3A shows the Kaplan‒Meier survival curves for this test set. The observed survival outcomes were consistent with the predictions of our model, thereby reinforcing its validity.

Multiple clinical factors, including age, sex, concomitant diseases, blood β2-MG, albumin, and G-band karyotype, are associated with the prognosis of multiple myeloma. There are multiple international prognostic assessment systems for MM that consider these factors, but their performance varies widely. To validate the prognostic value of the lncRNA signature using Kaplan‒Meier survival analysis and the Cox proportional hazards model, we constructed a nomogram based on multifactor models integrating clinical parameters and the lncRNA signature to predict MM prognosis (Fig. 3B).

### Identification of lncRNA functions and ceRNA network construction

**Fig. 4:**
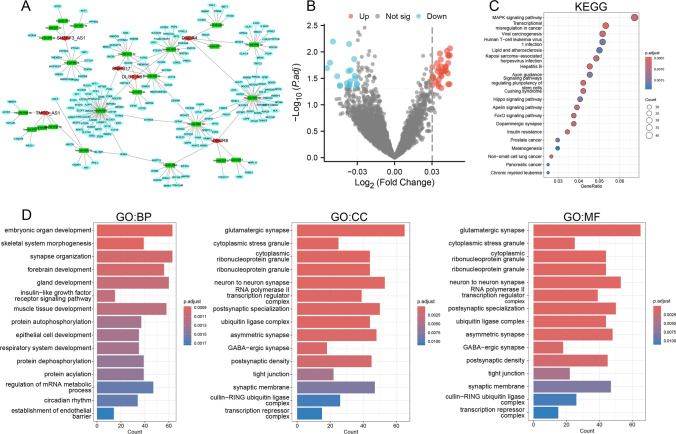
The ceRNA network of 6 lncRNAs and differentially expressed genes between the risk score groups. **A** The ceRNA network of 6 lncRNAs. **B** Differential gene expression in patients grouped according to lncRNA risk score. **C** KEGG enrichment analysis of differentially expressed genes. **D** GO analysis of differentially expressed genes

In this study, the 6 identified lncRNAs were entered into the miRDB database to predict their respective miRNA targets. The top 5 miRNAs corresponding to each lncRNA were incorporated into the ceRNA network. Next, predictions for the coding genes of these miRNAs were made, yielding a total of 2015 mRNAs. Figure 4A shows a comprehensive overview of the constructed triple-ceRNA (lncRNA–miRNA–mRNA) network. A total of 4922 pathways were enriched, and differentially enriched pathways are presented in a volcano plot; there were 53 upregulated pathways and 56 downregulated pathways (Fig. 4B). The top GO and KEGG pathway results are depicted in Fig. 4C, D; several enriched cancer-related pathways are highlighted and need to be further analyzed.

### Analysis of the correlations of the risk score with immunosuppression-related genes and immune cell infiltration

**Fig. 5:**
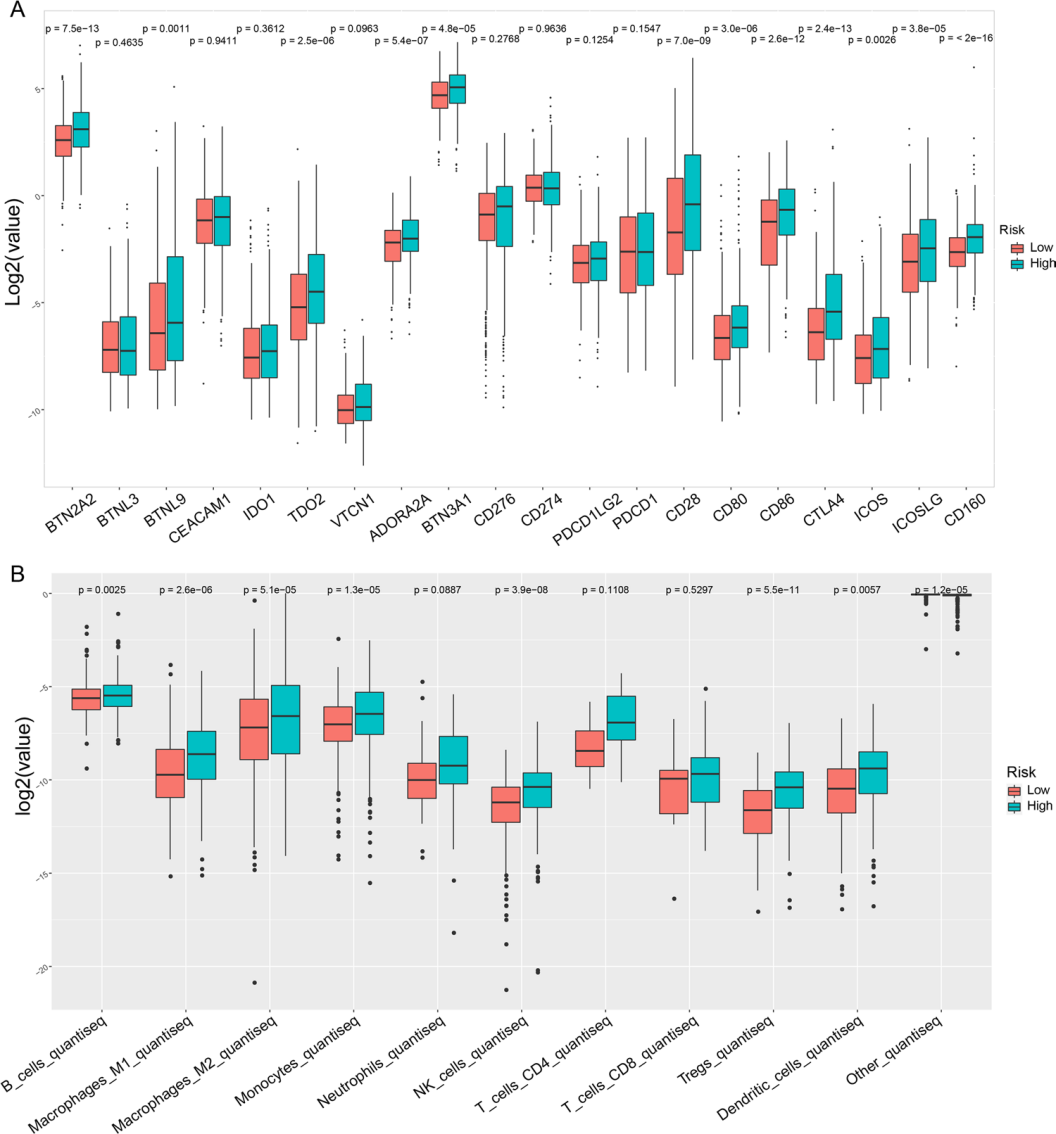
Correlation analysis of immune cells and immunosuppression-related genes based on the lncRNA risk model. **A** Correlation analysis based on the lncRNA risk model. **B** A few immune cell types correlated with the lncRNA risk score

The tumor microenvironment (TME) is infiltrated by many types of innate and adaptive immune cells whose immune-surveillance functions are often suppressed by multiple mechanisms. Tumor cells suppress this signaling by decreasing the activity of stimulatory immunoreceptors and increasing the activity of inhibitory immunoreceptors. Several inhibitory immunoreceptors, including but not limited to CD28, CD80, CD86, CTLA4, CD276, and CD274, have been identified and studied in cancer in recent decades. They are known as “immune checkpoints”, which refers to molecules that act as gatekeepers of the immune response. In this study, the MMRF cohort was divided into two groups according to the risk score of the lncRNA signature, and the expression levels of immune checkpoint genes in the two groups were analyzed. The results showed that the expression levels of multiple genes were also higher in the group with high scores, indicating that patients in the group with a poor prognosis had some suppression of tumor immunity (Fig. 5A).

To assess changes in immune cell subsets in patients in different risk groups, we used quanTIseq to investigate the potential regulatory effects of the lncRNA signature on the immune cell composition of the MM TME in the dataset MMRF. The quanTIseq deconvolution results revealed remodeling of the immune cell population (Fig. 5B). The changes included significant increases in the M1 and M2 macrophage, monocyte, NK cell, dendritic cell, B cell, and Treg cell fractions, suggesting the possible effects of the lncRNA regulatory network on immune cells are complex; for example, lncRNAs affect not only cancer-killing cells but also immune inhibitory cells such as Treg cells and M2 macrophages.

### Chemotherapeutic drug sensitivity analysis

**Fig. 6:**
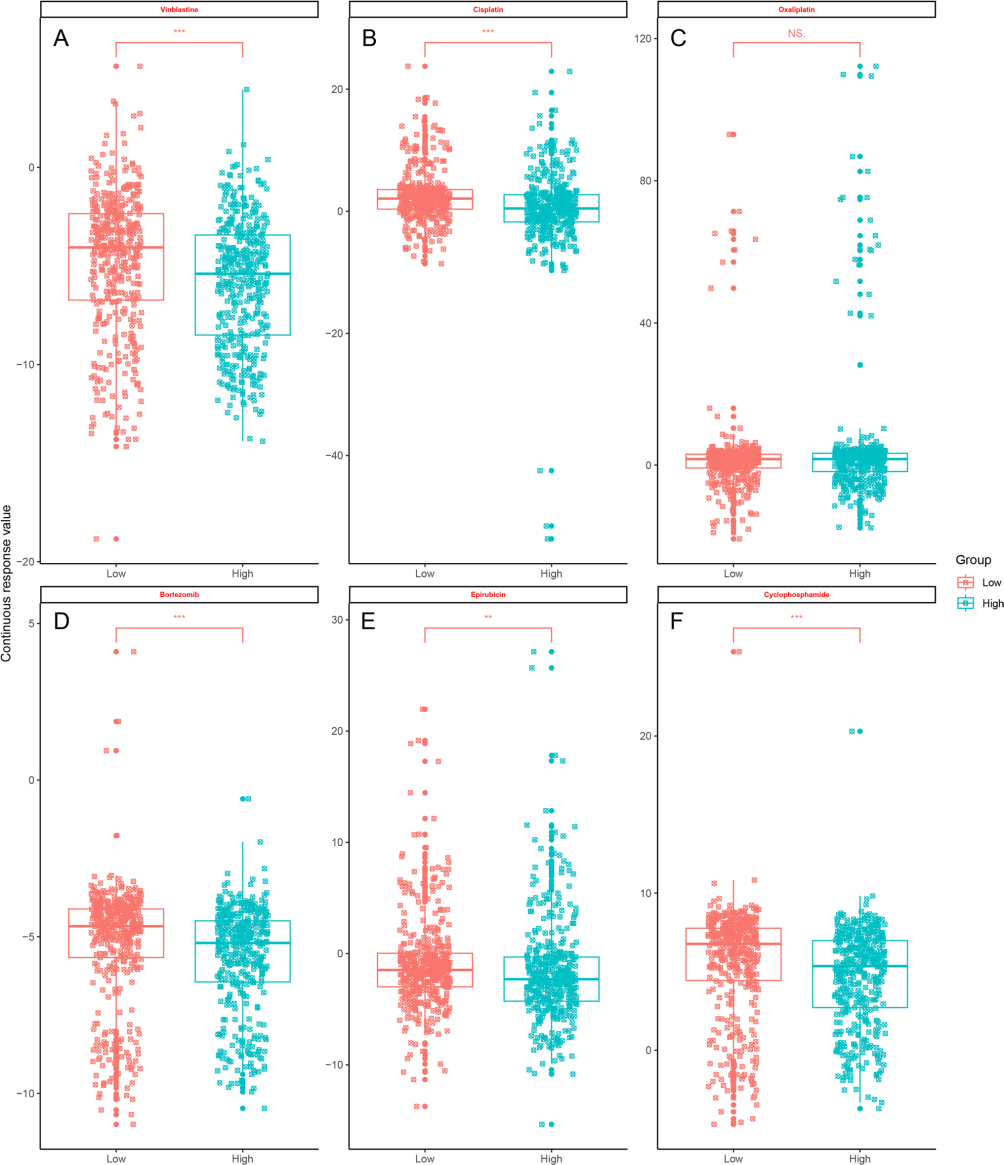
Chemotherapeutic drug sensitivity analysis based on lncRNA risk scores. **A**–**F** show the results of vinblastine, cisplatin, oxaliplatin, bortezomib, epirubicin and cyclophosphamide analysis

The chemotherapy resistance of malignant tumor cells is the current main challenge in tumor treatment, and new strategies are needed. The mechanism of tumor drug resistance is complex and involves multiple factors, such as abnormal intracellular drug transport, altered DNA damage repair, and inhibition of apoptosis. However, these findings are still insufficient to solve the problem of drug resistance in tumor cells, and it is necessary to explore in depth from new perspectives to elucidate the mechanism of drug resistance in tumors. The multiple biological functions and characteristics of lncRNAs have made them useful for studying the biological behaviors of malignant tumors and revealing the mechanism of chemotherapy resistance in malignant tumors by providing new ideas and methods. By applying our screened lncRNA risk assessment signature, patients in the MMRF cohort were divided into two groups, and oncoPredict was used to predict the sensitivities to MM chemotherapeutic agents for the two groups. The results showed that the high-risk group had significantly lower sensitivity to cisplatin, vincristine, epirubicin, cyclophosphamide, and bortezomib (Fig. 6), suggesting that these lncRNAs may be related to the poor prognosis of the high-risk group.

### Knockdown of TMPO_AS1 and SNHG17 significantly inhibited the proliferation and colony formation of NCI-H929 cells

**Fig. 7:**
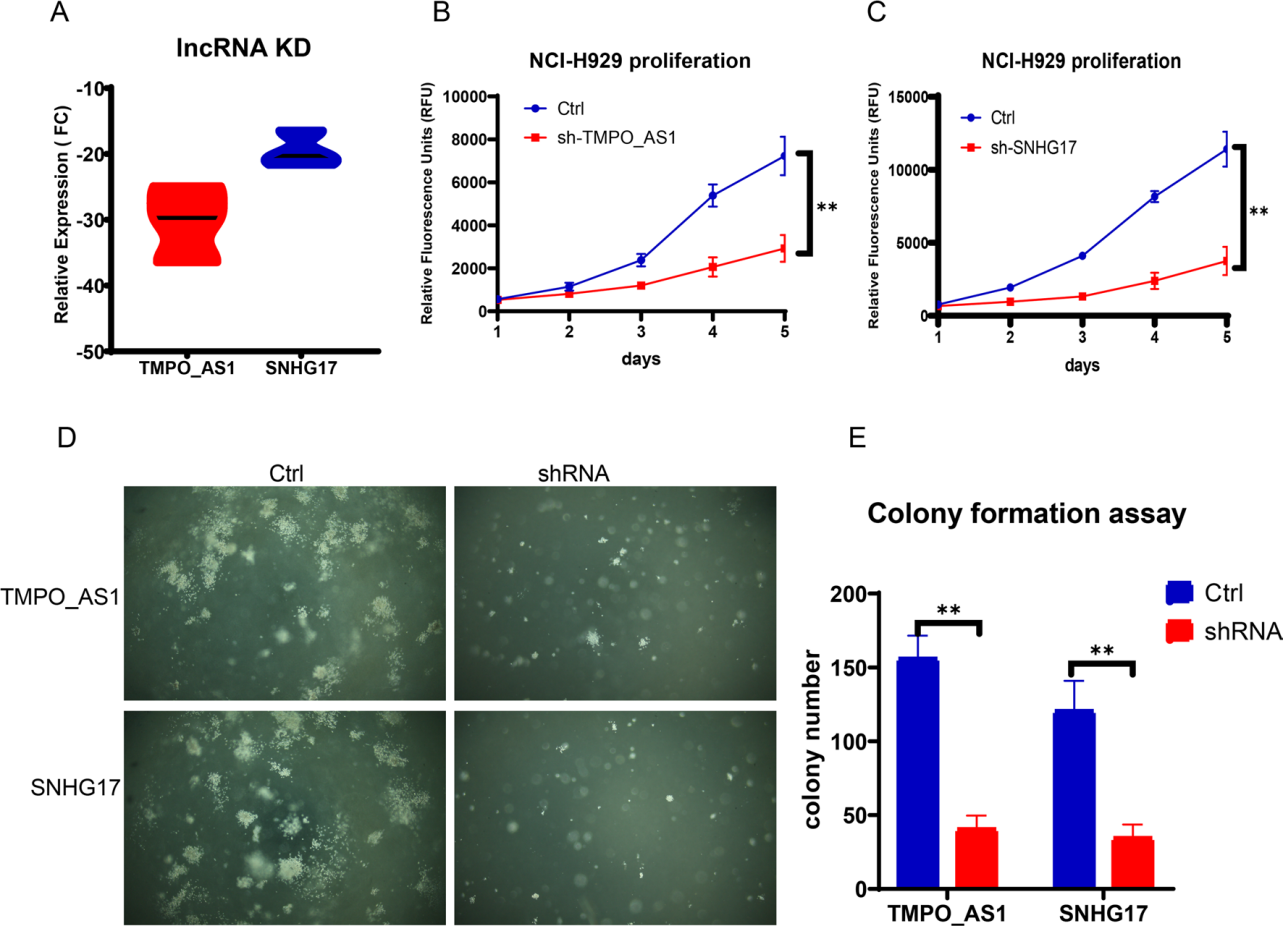
Knockdown of TMPO_AS1 and SNHG17 significantly inhibited the proliferation and colony formation of NCI-H929 cells. **A** shRNA was used to knockdown lncRNA expression in cells. **B**, **C** Knockdown of TMPO_AS1 and SNHG17 significantly inhibited the growth of NCI-H929 cells. **D**, **E** The colony formation of NCI-H929 cells was significantly inhibited by TMPO_AS1 or SNHG17 knockdown

To verify the effect of lncRNA levels in tumor cells obtained by our data analysis, we selected two lncRNAs with the largest calculated weights and previously reported in the literature [23, 24], TMPO_AS1 and SNHG17, as experimental subjects. shRNA lentiviral vectors were constructed according to the sequences, and then, NCI-H292 myeloma cells were infected. TMPO_AS1 and SNHG17 were knocked out by shRNA, after which the effects on the growth of tumor cells were observed. The results showed that the transcription levels of the two lncRNAs decreased significantly (Fig. 7A), and the proliferation ability of the tumor cells was significantly inhibited (Fig. 7B, C). A soft agar colony formation assay also demonstrated that the growth ability of NCI-H929 cells in soft agar was inhibited after TMPO_AS1 and SNHG17 were knocked out, and the number and diameter of the colonies were significantly reduced (Fig. 7D, E).

## Discussion

The role of lncRNAs in the pathogenesis of MM and their potential as biomarkers has recently garnered considerable attention. In our study, we conducted a comprehensive analysis of lncRNAs expressed in MM and identified 422 lncRNAs significantly associated with patient overall survival. Subsequent analysis using a robust likelihood-based survival model was used to filter the candidate pool to 15 lncRNAs with the lowest P values and then to six lncRNAs with the lowest Akaike information criterion (AIC) values. These six lncRNAs demonstrated predictive potential for 5-year overall survival in MM patients across both the training and validation sets, indicating their potential utility as prognostic biomarkers in clinical practice [25–28].

In addition to their role as biomarkers, lncRNAs are implicated as “hallmarks of cancer,” influencing early tumor onset and progression [25]. They may act as microRNA (miRNA) sponges in competing endogenous RNA (ceRNA) networks, influencing the formation and progression of multiple myeloma [29]. This ceRNA hypothesis suggests that transcriptional products sharing miRNA response elements (MREs) can regulate each other’s expression through miRNAs, thereby affecting gene regulation in cancer [30, 31]. In our study, a triple-ceRNA (lncRNA–miRNA–mRNA) network was constructed, and bioinformatics analysis revealed that six identified lncRNAs might function as ceRNAs to regulate genes involved in cancer-associated signaling pathways [32, 33].

The analysis of the expression levels of 20 immune checkpoint-related genes in MM, as derived from the MMFR dataset, provides critical insights into the immunological interactions and potential therapeutic targets of this disease. Elevated expression levels of genes such as BTN2A2, BTNL9, TDO2, ADORA2A, BTN3A1, CD28, CD80, CD86, CTLA4, ICOS, ICOSLG, and CD160 have significant implications for understanding disease progression and treatment response. In particular, high expression levels of CD28 and CD86 have been identified as poor prognostic markers in myeloma patients, indicating their crucial role in disease survival and progression mechanisms [34]. Furthermore, ICOSL, another gene in this list, is an early differentiation marker in certain cell maturation pathways, suggesting its potential role in the development of MM [35]. The expression of these genes indicates their involvement in leukemic processes and tumor progression, highlighting their significance in the context of MM. Elevated levels of these genes could be associated with disease progression, treatment response, and possibly overall prognosis. This information is valuable for understanding the molecular underpinnings of MM and could aid in developing targeted therapies or prognostic models for this type of cancer.

The quanTIseq method utilizes a deconvolution algorithm to analyze RNA sequencing data from bulk tumor samples, which allows for the estimation of the composition of various immune cell types within the tumor microenvironment. This approach is pivotal for elucidating the intricate landscape of the tumor immune landscape, providing insights into tumor immune evasion strategies and potential immunotherapeutic targets. Elevated levels of B cells, macrophages (both M1 and M2 types), monocytes, NK cells, Treg cells, and dendritic cells in the context of MM have diverse implications. B cells may contribute to the local immune microenvironment by producing antibodies that can affect tumor growth. The presence of M1 macrophages, which are typically associated with proinflammatory responses, could indicate a tumor-suppressive environment, while the presence of M2 macrophages, which are linked to anti-inflammatory responses, may indicate the presence of a tumor-promoting microenvironment that facilitates tumor growth and survival [36, 37]. Monocytes can differentiate into various cell types and may influence tumor progression and the response to treatment [38]. NK cells are critical for the direct killing of tumor cells, and their elevation might reflect an active immune response against MM cells [39]. Treg cells, known for their immunosuppressive functions, can contribute to immune evasion by tumors [40]. Finally, dendritic cells are essential for antigen presentation and the initiation of adaptive immune responses; their presence might indicate an active immune surveillance environment [41]. Together, these cell types make up a complex network that could profoundly influence disease progression, treatment responses, and overall patient outcomes in MM.

The ongoing challenge in cancer therapy is overcoming chemotherapy resistance, a multifaceted issue involving numerous cellular mechanisms such as aberrant drug transport, DNA repair alterations, and apoptosis inhibition. Despite extensive research, the current understanding is insufficient to fully address this resistance, highlighting the need for innovative approaches to unravel these complex mechanisms. LncRNAs have emerged as key players in the biological behaviors of malignant tumors, including in the treatment of chemotherapy resistance. Their diverse functions and characteristics offer fresh perspectives and methodologies for investigation. In the context of the results from the MMRF dataset, the utilization of a lncRNA risk assessment signature to categorize patients represents a novel stratification approach. Analysis with the oncoPredict tool revealed a significant difference in chemotherapeutic sensitivities between the high-risk and low-risk groups, particularly for agents such as cisplatin, vincristine, epirubicin, cyclophosphamide, and bortezomib. This suggests that lncRNAs associated with the high-risk group may contribute to the observed resistance and poor prognosis [42]. This finding aligns with findings from other studies in which lncRNAs were implicated in drug resistance mechanisms, either by affecting drug efflux, altering cell survival pathways, or modifying the tumor microenvironment [13, 15]. Therefore, targeting these lncRNAs could reverse resistance and improve patient outcomes, offering a promising direction for future research and therapeutic development [43].

The lncRNAs TMPO-AS1 and SNHG17 have emerged as significant players in cancer biology. TMPO-AS1, which is upregulated in various cancers, such as breast and esophageal squamous cell carcinoma, promotes cell proliferation and metastasis, potentially through mechanisms involving miRNA sponging and modulation of gene expression, such as TMPO. Similarly, SNHG17, a member of the SNHG family, is highly expressed in multiple cancers, including melanoma and lung adenocarcinoma. It is implicated in tumor cell proliferation, migration, invasion, and drug resistance and often acts as a competing endogenous RNA (ceRNA) to influence miRNA activity. Both lncRNAs are associated with poor prognosis and may serve as potential biomarkers for cancer diagnosis or targets for therapeutic intervention. The roles of lncRNAs in cancer progression underscore the complexity of gene regulation in oncogenesis and the potential of lncRNAs as novel targets in cancer therapy. Our experiments confirmed that lncRNAs play important roles in myeloma cell proliferation and progression, providing a basis for their clinical application in prognosis [10, 15].

Taken together, these results suggest that the identified lncRNAs may play a critical role in signaling pathways associated with tumorigenesis, regulation of immune cell action, and chemotherapy resistance, thereby affecting the prognosis of MM, and have potential as prognostic biomarkers in MM clinical practice. However, despite these advancements, the study has limitations. The results are primarily based on bioinformatics analyses and lack validation through in vitro or in vivo experimentation. Future research should explore the biological functions of these lncRNAs, particularly their impact on cell proliferation and apoptosis, and validate their utility as prognostic biomarkers.

## Conclusion

This study highlights the significance of lncRNAs in MM, particularly their potential as biomarkers. An extensive analysis revealed 422 lncRNAs linked to overall survival, with six key lncRNAs demonstrating strong predictive value for 5-year survival. These lncRNAs also function within competing endogenous RNA (ceRNA) networks, potentially influencing cancer progression and gene regulation. Additionally, the analysis of 20 immune checkpoint-related genes in MM provides valuable insights into the immunological dynamics of this disease and prospective therapeutic targets. The analysis of RNA sequencing data by the quanTIseq method further revealed the tumor immune landscape, underscoring the diverse roles of various immune cells in MM.

### Supplementary Information

Supplementary Material 1.

Supplementary Material 2.

## Data Availability

All datasets were sourced from publicly accessible databases and published studies. All the papers cited in this manuscript can be found on PubMed or Google Scholar. Additional data generated during this study, as well as the code used, can be obtained upon request from the corresponding authors.
